# An apta-aggregation based machine learning assay for rapid quantification of lysozyme through texture parameters

**DOI:** 10.1371/journal.pone.0248159

**Published:** 2021-03-08

**Authors:** Manoharan Sanjay, Kumar Gaurav, Maria Jesus Gonzalez-Pabon, Julio Fuchs, Susan R. Mikkelsen, Eduardo Cortón

**Affiliations:** 1 Biosensors and Bioanalysis Laboratory (LABB), Department of Biological Chemistry and IQUIBICEN-CONICET, Exact and Natural Sciences Faculty (FCEN), University of Buenos Aires (UBA), Buenos Aires, Argentina; 2 Department of Biosciences and Bioengineering, Indian Institute of Technology Guwahati, Guwahati, Assam, India; 3 Department of Chemistry, University of Waterloo, Waterloo, Ontario, Canada; University of Pennsylvania, UNITED STATES

## Abstract

A novel assay technique that involves quantification of lysozyme (Lys) through machine learning is put forward here. This article reports the tendency of the well- documented Ellington group anti-Lys aptamer, to produce aggregates when exposed to Lys. This property of apta-aggregation has been exploited here to develop an assay that quantifies the Lys using texture and area parameters from a photograph of the elliptical aggregate mass through machine learning. Two assay sets were made for the experimental procedure: one with high Lys concentration between 25–100 mM and another with low concentration between 1–20 mM. The high concentration set had a sample volume of 10 μl while the low concentration set had a higher sample volume of 100 μl, in order to obtain the statistical texture values reliably from the aggregate mass. The platform exhibited an experimental limit of detection of 1 mM and a response time of less than 10 seconds. Further, two potential operating modes for the aptamer were hypothesized for this aggregation property and the more accurate mode among the two was ascertained through bioinformatics studies.

## Introduction

Agglutination is a term that is generally confined to antibody-antigen interactions that result in an interlinked mass of antigens and antibodies. It is commonly used in ABO blood typing where antibodies attached to latex particles are mixed with the blood sample. When these antibodies crosslink with a polyvalent antigen it results in agglutination or precipitation of particles [1, 2]. Besides blood-typing, agglutination techniques were also historically used for classifying beta-hemolytic streptococci based on antigenic differences through anti-sera [3]. This is an example of a direct/active agglutination assay, since the Streptococci have an inherent tendency to agglutinate. When the aggregate visualization is enhanced by the attachment to inert latex beads it qualifies as passive agglutination assay as the agglutination of the beads indicates the presence of an antibody binding to some other antigen [4]. Agglutination assays are inexpensive, since agglutination can be detected visually. Additionally they are simple, rapid, often requiring only a few minutes and offer a high degree of sensitivity [5]. A plethora of assays based on antibodies have been explored. A few such assays include the latex beads-facilitated antibody agglutination assay for *Tritrichomonas foetus* described by Schaut *et al*., [6], measurement of von Willebrand factor antigen through a latex agglutination test illustrated by Mahat *et al*. [7], and a microfluidic immuno-magnetic agglutination assay for detection of dengue virus NS1 antigen published by Alejo-Cancho *et al*., [8].

However, the main disadvantage of this technique along with its qualitative/semi-quantitative nature is its use of antibodies [9]. The involvement of antibodies renders these assays temperature-sensitive and drives up the cost per test due to the high costs involved in antibody production and purification [10, 11]. In that context, new-generation of affinity molecules like aptamers are being widely preferred due to their effectiveness and robustness [12]. Aptamers have already found application in several bioassay modalities such as Quartz crystal microbalance [13, 14], Cantilever-based biosensing [15, 16], electrochemistry [17], Surface plasmon resonance [18, 19] and spectrophotometric assays [20].

The usage of aptamers in aggregation assays is not widely explored because of the monovalency of the aptamer towards the analyte. Agglutination assays strictly require both the analyte and the ligand to be polyvalent. Unlike the bivalent IgA antibody or the polyvalent IgM antibody, aptamers possess only a single binding site. Further, the analyte in question, at most instances possesses only a single binding site. This is generally circumvented by coupling aptamers to microbeads as it imparts polyvalency to the beads and facilitates agglutination. Polystyrene micro/nano beads were utilized to illustrate the same by Kim *et al*., and the agglutination mass was quantified through a simple light scattering assay [21]. In the study by Uddin *et al*., aptamer coated magnetic beads were used for agglutination to facilitate an optomagnetic readout [22]. The problem of antigen polyvalency is often overlooked by opting for inherently polyvalent analytes. Cells are often the best candidates as their exteriors are covered with numerous antigens of the same kind thereby rendering them polyvalent. For instance, the numerous lipophosphoglycan molecules that coat the surface of *Tritrichomonas foetus* have been capitalized for the development of an agglutination assay [6]. Hence, agglutination assays for other kinds of analytes without polyvalent beads remains to be researched.

**Fig 1:**
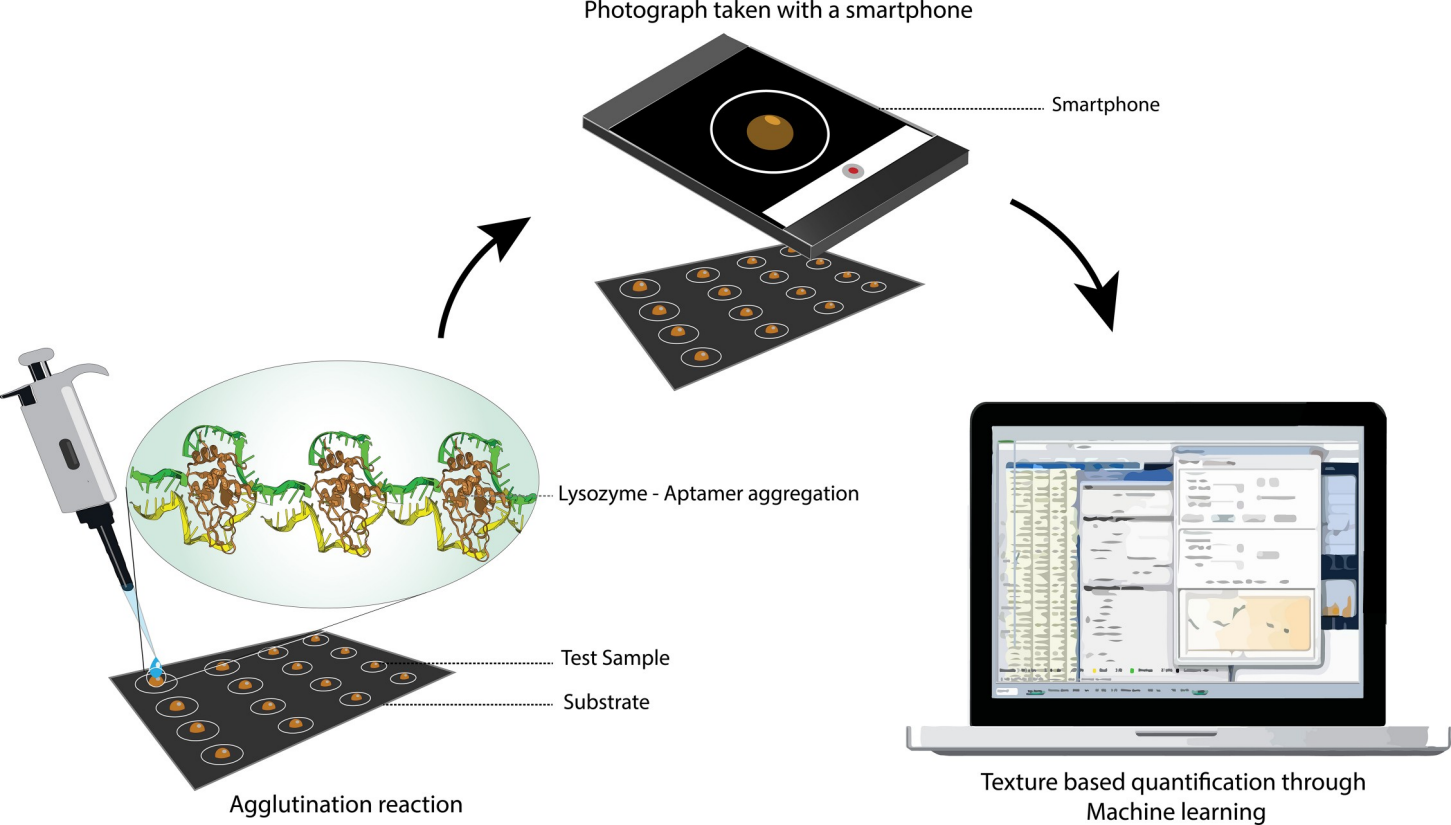
Steps involved in the quantification of the apta-aggregation assay.

Turbidimetry and optomagnetism-based detection are the widely used techniques for quantification of agglutination assays. Turbidimetry involves the process of measuring the loss of transmitted light intensity due to the scattering effect of particles suspended in it and it is determined through a microtiter-plate reader [23]. The optomagnetic detection technique employs a Blu-ray optical pickup unit as the excitation element, a photodetector as the sensing element and an AC magnetic field to provide the excitation energy. When the magnetic beads agglutinate, they exhibit optical anisotropy upon application of the oscillating magnetic field. Due to the large size of these agglomerates, the dynamics of this process is shifted to frequencies well below the Brownian relaxation frequency of the individual beads and this is interrogated through a photodetector [24]. These large, expensive and complex quantification equipment confine aggregation assays to lab-scale measurements despite the simplicity of these reactions. In that context, there is a need for inexpensive quantification platforms and smartphones with their state-of-the-art components and computation power coupled with their easy availability make for ideal candidates. Hence, we present here a novel apta-aggregation assay, where Lys containing samples are placed as droplets on a substrate followed by dropping the anti-Lys aptamer solution. Lys was quantified by employing a neural network trained with texture parameters and area of the aggregate mass obtained through a photograph taken with a smartphone and without the use of polyvalent microbeads as shown in Fig 1. The Ellington *et al*., anti-lys aptamer has been used for the purpose and its efficiency and specificity has been verified extensively through different transduction mechanisms in literature [25–30].

A direct way to quantify aggregation could be through histogram analysis of captured photos but its reproducibility issues have raised major concerns in the past [31]. Alternatively, establishing a relationship between aggregate area and Lys concentration for quantification could be a straight-forward approach as well. However, this is more complicated than it sounds, as the area can be greatly affected by the way in which the aptamer solution is dropped over the Lys droplet such as the trajectory, height from which it is dropped and angle of the drop. Whilst controlling these parameters is impossible without automation, a more logical way forward is to exploit the aggregate area-texture relationship. For a constant concentration of aptamer and lysozyme, the texture of its aggregate mass changes with the aggregate area due to variabilities in the aptamer dropping i.e., an aggregate mass of a small area will be coarse and compact while a large area aggregate will be smooth and diffused. Hence, this interplay of texture and aggregate area has been capitalized for this assay. The use of free aptamers in solution to detect a wide range of protein analytes ranging from C-Reactive Protein to Plasmodium Lactate dehydrogenase has been reported widely in literature via gold nanoparticle (GNP) aggregation assays [32–34]. Lysozyme has also been previously quantified using an assay with gold nanoparticles and anti-lys 25 base pair aptamer by Yao *et al*., [35]. The use of GNPs adds to the cost and complexity, all the while suffering from the previously mentioned shortcomings of histogram-based quantification. However, the prior existence of these assays including the one with lysozyme by Yao *et al*., verified the feasibility and laid the foundation for the texture-based quantitative assay devoid of Gold-nanoparticles and polyvalent beads.

The texture of an image can be defined as the spatial grouping of color or intensities in an image or selected region of an image. A statistical method for studying texture is through gray-level co-occurrence matrix (GLCM), which considers the spatial relationship of pixels. The GLCM functions characterize the texture of an image by determining the frequency of occurrence of pairs of pixels with specific values and in a specified spatial relationship in an image to create a GLCM. The statistical functions are extracted from this matrix which act as indicators of the image texture [36, 37]. Texture parameters extraction is often coupled with machine learning and has been widely used in remote sensing [38] and even for cancer classification or diagnosis from computer tomography, ultrasound and MRI images [39–41].

Aggregation without polyvalent microbeads was made possible through the unique aggregation property demonstrated by the anti-Lys aptamer uncovered by this study. The requirement of polyvalency of both aptamer and protein for aggregation assays has been highlighted previously [20]. In that light, the ability of this aptamer to form aggregates on exposure to Lys made us hypothesize two modes-of-interaction Mode.1- Lys has two binding sites for the aptamer and the aptamer has two binding sites for Lys or Mode.2- Lys has two binding sites for aptamer but aptamer does not contain two binding sites for Lys, instead it binds to adjacent Lys-bound aptamers through Watson-Crick complementary base pairing. In order to unravel the mode of operation, bioinformatics tools such as RNA composer and HDock were employed. This part of the study provided insights into the inner workings of all aptamer-analyte interactions in general and can potentially prove useful for future aptamer design and development.

## Materials and methods

### Aggregation reaction

Lysozyme from hen’s egg white dissolved in ultrapure water (= 18.2 MΩ) from a Millipore-MilliQ system at different concentrations was used as the test sample. Lysozyme from chicken egg white and bovine serum albumin (BSA), were purchased from Sigma–Aldrich. The DNA-anti-Lys aptamer developed by Ellington *et al*., (5′-ATC TAC GAA TTC ATC AGG GCTAAA GAG TGC AGA GTT ACT TAG-3’) was purchased from Integrated DNA Technologies, Inc. [42]. The anti-Lys aptamer was heated at 70°C for 3 minutes before exposure to Lys. This is essential for preserving the structural capabilities and increasing the efficiency of analyte capture [26]. Two sets of Lys test samples were prepared, a high concentration set “A” (25 mM- 400mM) and a low concentration set “B” (1–20 mM). The Set-A assay sample volume was fixed to 10 μl and the B set volume to 100 μl. Lower concentrations (set “B”) produced no visible aggregates or micro aggregates that were not resolvable by the smartphone camera when using 10 μl sample volume. Hence by choosing a higher sample volume among lower analyte concentrations, more lysozyme molecules were available for aggregation and therefore a visually resolvable aggregate mass, increasing the concentration range that can be measured. These samples were placed on a reaction substrate using a micropipette and studied as triplicates. The reaction substrate was made by printing an image containing 35 circular reaction areas on a black background on a generic photographic paper as seen in Fig 1. The hydrophobicity of the photo paper was evaluated as per Chen *et al*., [43]. The liquid-substrate contact angle was found to be 94.686° indicating that it is hydrophobic [44]. This hydrophobic property along with the surface tension of droplets provided resulted in reaction droplets of fairly similar geometry. The test samples were then exposed to heat treated 3 μl aptamer solution of 25 nM concentration followed by waiting for approximately a minute after which the aggregates were observed. The 3 μl aptamer solution volume was finalized following an experiment to determine the effect of aptamer volume on aggregation intensity. In short, five 10 μl Lys reaction droplets of 25 mM concentration each were exposed to aptamer volumes between 1 μl to 5 μl and made up to 15 μl followed by studying their individual aggregation intensities. All the reactions were carried out directly on a lab bench at 20°C and a relative humidity of 70–90%. The results from the triplicates, formed the training data for the neural network. The test samples to evaluate the neural network were made along with the training triplicates with set concentrations of Lys. Further, the occurrence of aggregation exclusively to the Anti-lys aptamer-Lysozyme (Apta-Lys) samples was confirmed with a control (10 μl of 25 mM lysozyme alone), Negative control (25 mM BSA+ Anti-Lys aptamer) and the test condition (3 μl of anti-lys aptamer with 10 μl of 100 mM lysozyme).

### Texture analysis and machine learning

The aggregates were then photographed using Oppo CPH1919 smartphone with a 48 Megapixel pixel-binning CMOS sensor (Sony IMX586) and analyzed using Image-J in a computer. These images were first converted into 8-bit and then their aggregate areas (Area) were measured via free-hand selection. Secondly, their texture was analyzed using GLCM texture plug-in V 0.4 developed by Julio E. Cabrera [45]. Various statistical parameters of the GLCM of the image such as Angular Second Moment (ASM), Inverse Difference moment (IDM), Entropy, Contrast and Correlation were provided by the plug-in. Triplicates were made and their statistical texture values along with the area of the aggregates were loaded as the training data for the neural network with a 6–1 architecture. Neural networking was performed by NeuroXL Predictor, a neural network-based add-in for Microsoft Excel developed by OLSOFT and is capable of advanced data forecasting, classification and clustering [46, 47]. The trained neural network was then tested with the data from a new set of Lys samples of pre-determined concentrations.

### Bioinformatics analyses

The mode of the aptamer can be deduced through docking studies for which the 3-D structure of the aptamer has to be first predicted. Currently, there are no tools for direct DNA 3-D structure prediction while direct tools for RNA structure prediction exist. This problem has been previously circumvented through an ingenious ssRNA-ssDNA conversion strategy as illustrated by Jeddi *et al*., and Alshaer *et al*., [48, 49].

A similar strategy as it was previously detailed described [48, 49] to model the 3-D structure of the aptamers has been implemented here and it involves the following steps- i) generation of ssDNA secondary structure through RNAfold webserver [50] ii) construction of equivalent 3D ssRNA models using RNA Composer [51] iii) translation of 3D ssRNA models into ssDNA models through manual modification of the hydroxyl (OH) group at the 2’- Carbon of the ribose with a hydrogen atom and replacing all thymine molecules with uracil. and iv) finally refining the 3D ssDNA structures through energy minimization using a CHARMM force field and 10000 steps of gradient energy minimization method using NAMD software [52, 53]. The docking simulation was performed between the predicted aptamer and the X-ray structure of the Lysozyme obtained from RCSB PDB database (PDB ID: 1DPX, Resolution: 1.65 Å) using HDOCK online server and the best pose was considered for all further studies [54]. In brief, one minimized aptamer was docked with lysozyme to get Lys-Apt models. The top 10 Lys-Apt models were re-docked with another aptamer to get Apt-Lys-Apt models. The top 10 models of each Apt-Lys-Apt model were analysed (100 models in toto). The model with the highest dock score without congruent binding sites was considered for further studies.

Further, in the event of Mode 2 (one bound aptamer hybridizing with neighbouring aptamer) being true, a strategy was devised to probe its probability by estimating the dock score of the hairpin formed by the free terminals of the Lys-bound aptamer using VFold2D and the ΔG value of the neighbour strand hybridization, if any using VFoldCPX server [55]. These servers do not provide DNA interaction data and hence the output from these servers were to be converted later to DNA manually in PyMOL [56].

Finally, the 3-D structure of the polymeric complex was to be visualized through the following steps—i) Acquiring the secondary structure of neighbour aptamer interaction using VfoldCPX. Since the input of two single strands is not allowed and the energetics of RNA-RNA interaction are similar to intra-molecular RNA folding, the potential hybridized strands were concatenated with non-sequential residues (AAA) and the same RNA folding algorithm as in Vfold2D server was to be utilized ii) Obtaining its tertiary structure by inputting Vfold2D output to RNAcomposer iii) Deletion of the non-sequential residues from both the 3D and 2D outputs iv) Manual conversion of all the outputs to DNA [55, 57–61]. It was to be followed by manually coupling two Aptamer-Protein-Aptamer complexes with the obtained hybridized aptamer ends to construct the probable structure of the polymeric complex. Finally, any non-specific interactions between the two aptamers bound to the lysozyme was probed by manually deleting the lysozyme from the complex and detecting the potential interactions using PPCheck webserver [62].

## Results and discussion

### Aggregation assay

**Fig 2:**
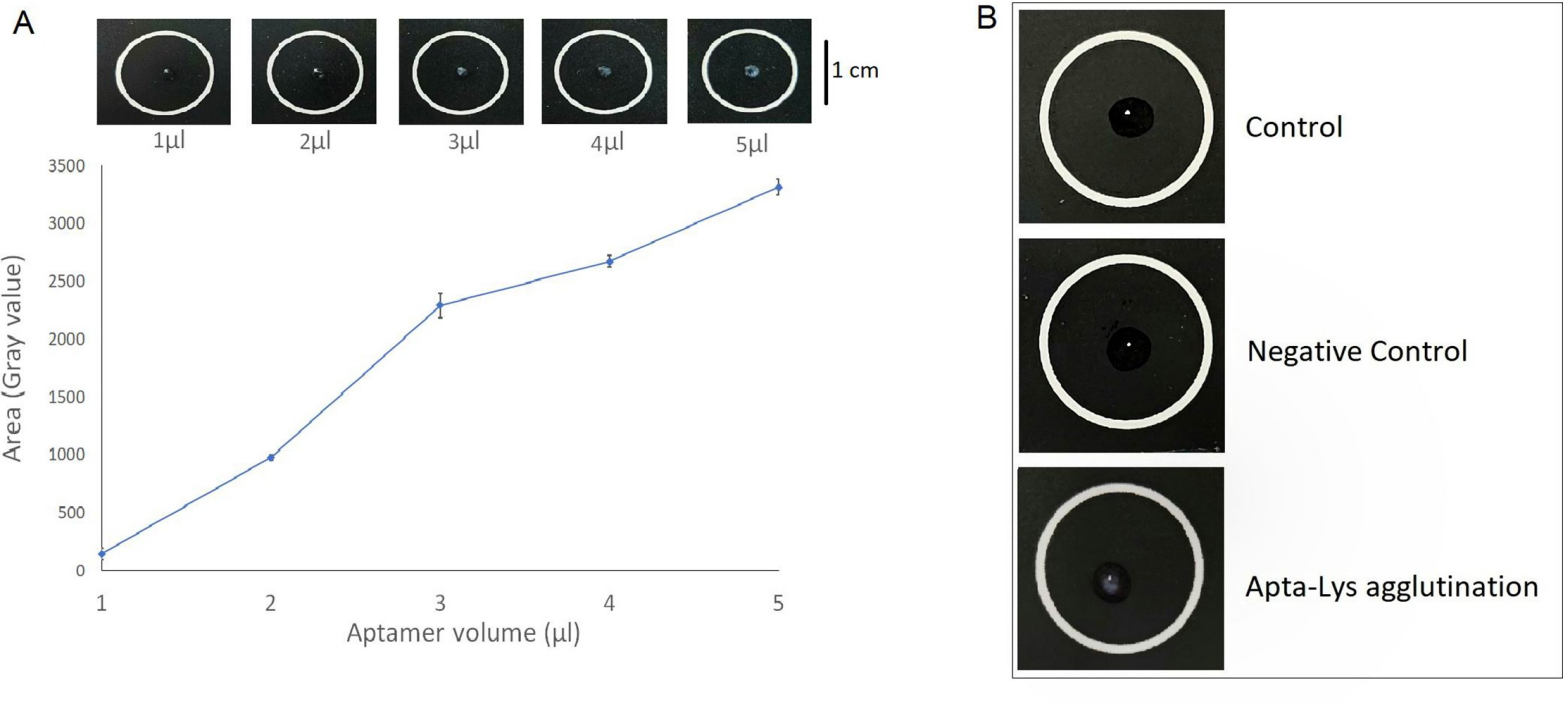
Aggregation assay. A) Impact of aptamer volume on aggregate area B) Test to confirm the exclusivity of aggregation to Anti-Lys aptamer and lysozyme.

The occurrence of aggregation exclusively to Apta-Lys samples was confirmed by comparing the texture from a control, negative control and test condition as shown in Fig 2B. There was no observable texture change in the controls whereas the test condition revealed an aggregate mass. Then the effect of aptamer concentration on aggregation intensity was examined. The aggregation area was found to have an almost linear relationship with the aptamer quantity and visually the aggregation intensity was also higher with high volume, with the 5 μl droplet volume showing the highest aggregation area. However, the 3 μl droplet was experimentally shown to produce a high volume to aggregate area ratio thereby making it an efficient option as illustrated by Fig 2A; at S4 Fig in S1 File the characteristic and LOD in order to determine the minimal amount of aptamer needed to perform one analysis in this condition is also shown. Therefore, the 3 μl aptamer volume was chosen for all further experiments.

Additional experiments were conducted to evaluate the dose response and it was found that lower concentrations of Lys produced barely observable aggregation. Hence the sample volume was increased to 100 μl, which in turn increased the number of Lys molecules available for the reaction. This formed the rationale behind the two sets of experiments namely, Set-A and Set-B. Both Set-A and Set-B, exhibited higher aggregation intensity with increasing concentrations. However, the area of aggregation varied widely. The Set-A displayed dense, increasingly intense, larger area aggregates in higher concentration samples because there are progressively more Lys molecules for the same volume to aggregate. In contrast, Set-B produced diffuse, less intense, larger area aggregates for low concentration and dense, small-area aggregates for high concentrations as portrayed by Fig 4. This can be attributed to the higher sample volume of Set-B. At 100 μl volume, low concentration samples are sparsely populated with Lys molecules which may mean that the aptamers, when dropped over the samples have to travel further in order to find their Lys molecules and aggregate. This may lead to thin or disjointed chains of Apta-Lys that precipitate thereby producing hazy, large area aggregates.

**Fig 3:**
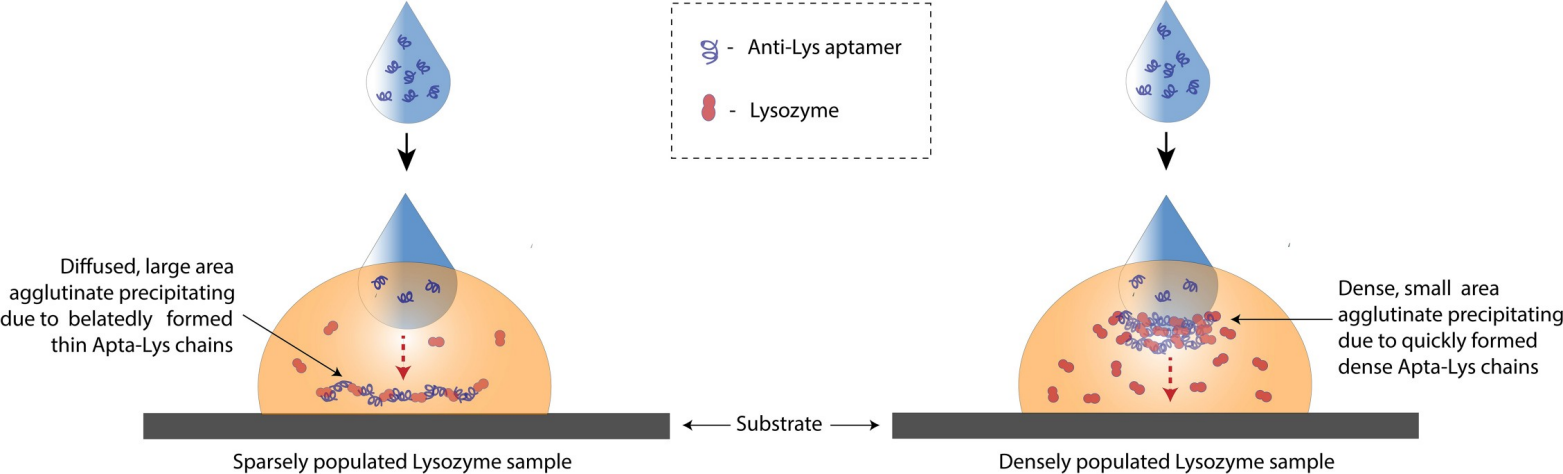
Formation of aggregates. Left, in sparsely populated (low concentration) and right, densely populated (high concentration) lysozyme samples.

**Fig 4:**
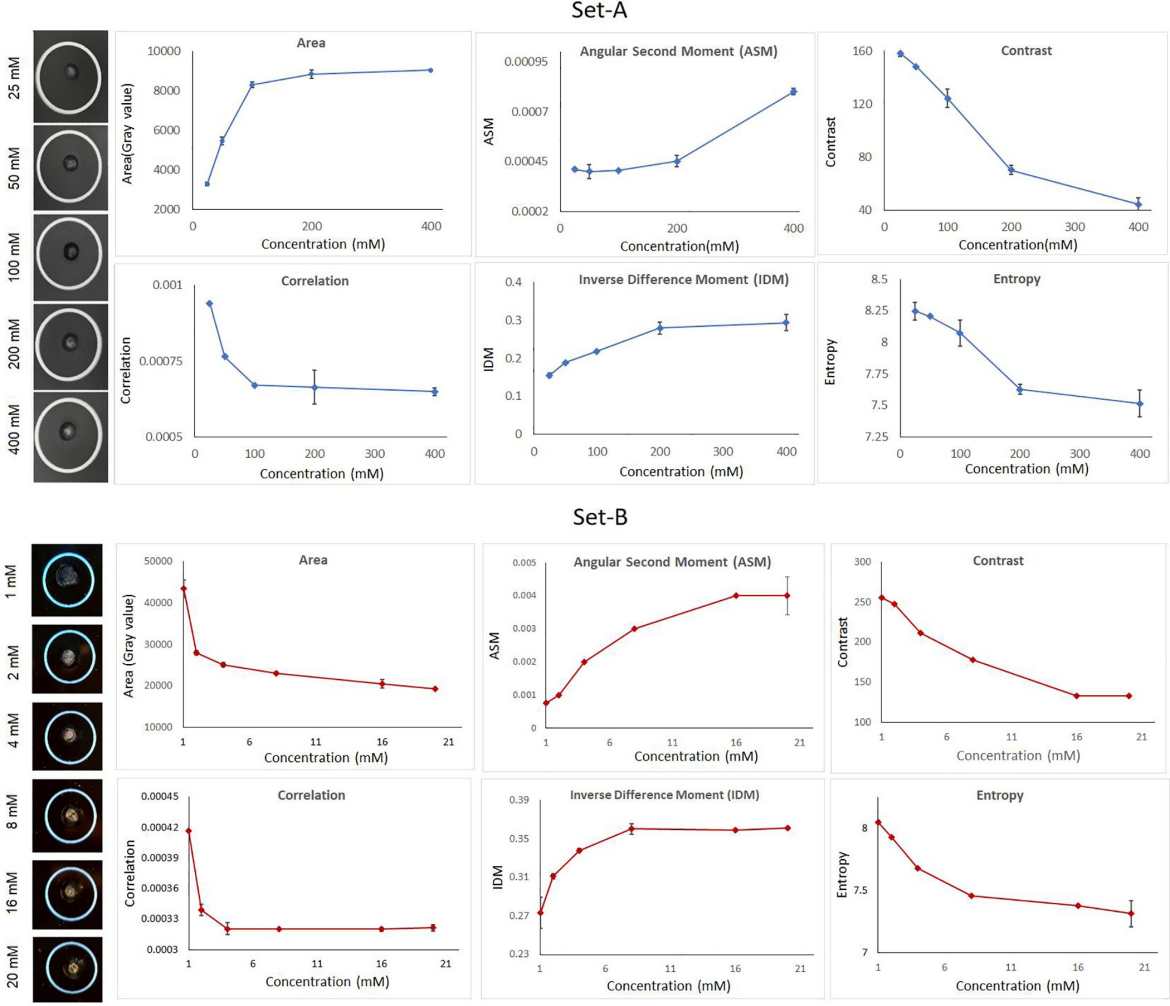
Texture parameters extracted from the aggregate masses.

On the other hand, a high concentration sample is densely populated with Lys and hence the aptamers have to travel less to find their Lys molecules which means that highly dense Apta-Lys chains are formed quickly and precipitate without much dispersion. This potential explanation for the phenomenon is illustrated in Fig 3. In brief, the lower concentrations-higher sample volume of Set-B and higher concentrations-smaller sample volume of Set-A led to a clear distinction in the aggregation dynamics. Though the area varied, increasingly intense masses were observed with high concentrations among both the sets and these masses seemed to exhibit a similar textural statistics trend as seen in Fig 4; some analysis about the sensitivity of each parameter shown is presented in S1 and S2 Tables in S1 File.

**Fig 5:**
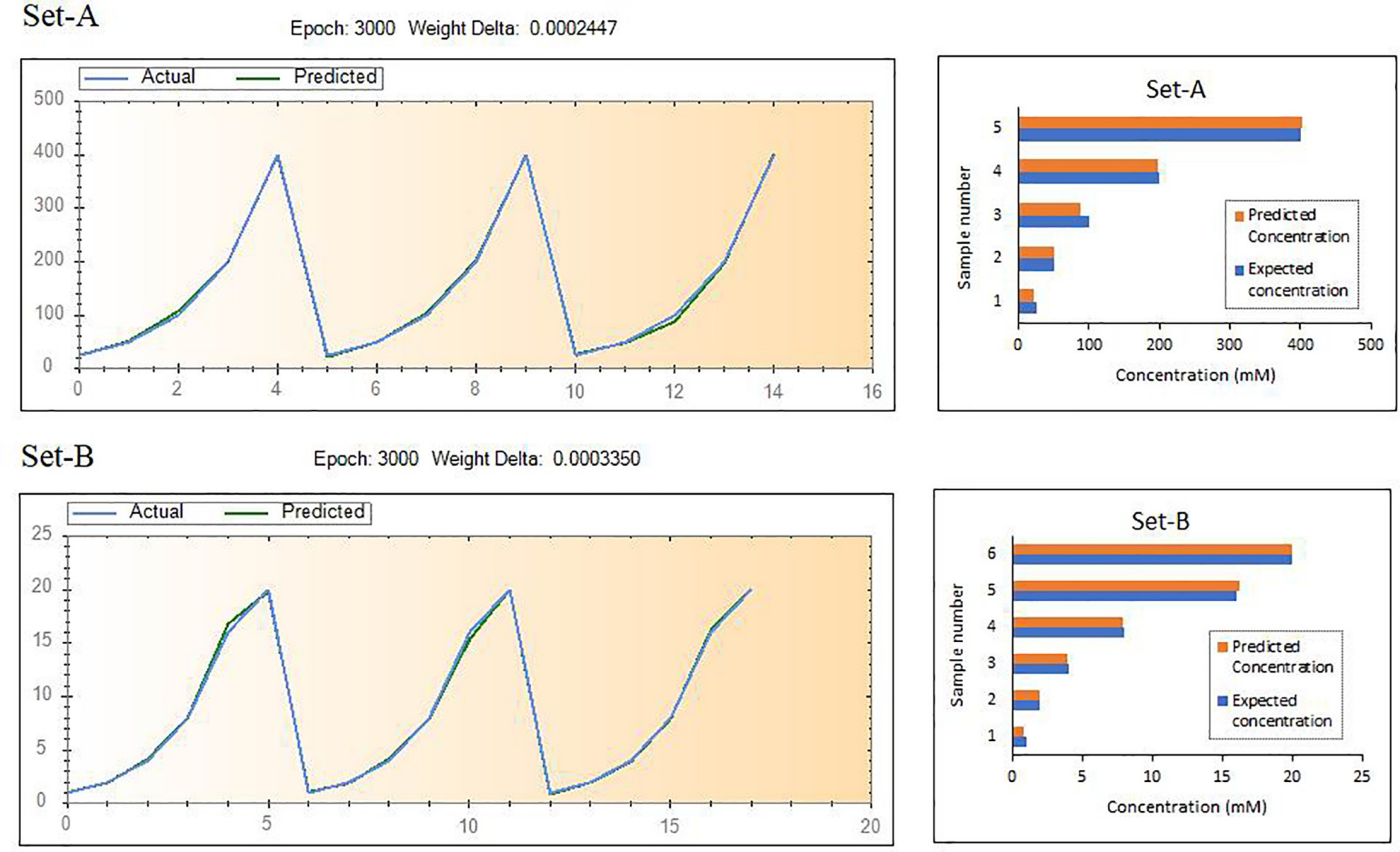
Trained model and the deviation of the predicted results from the expected values.

The angular second moment (ASM) is a measure of homogeneity of the texels while contrast is a measure of the amount of local variations existing in an image. Correlation represents how a reference pixel is related to its neighbour pixel, and can have values between -1 (perfectly correlated) to 1 with a value of 0 representing no correlation and masses with considerable amounts of linear structures can project high correlation. Inverse difference moment (IDM) is the measure of local homogeneity and increases with the quantity of linear structures in an image. High entropy usually implies elevated level of disorder and disorganization [36, 63–65]. In brief, ASM and IDM are indicators of homogeneity while contrast, correlation and entropy are indicators of chaos in the image. The trends of these parameters of both the sets indicated that increasingly homogenous and less chaotic masses are produced with higher concentrations as depicted by Fig 4. and two separate training models for Set- A and for Set-B were used for the neural network. The trained neural networks were used to predict the concentrations of the test set through the texture parameters extracted from a predetermined set of concentrations as mentioned in the previous section. The learning process of the model and the deviation between the predicted values and expected values of the test set are shown in Fig 5. On the whole, the efficiency of the prediction process was found to be approximately 96.365%, indicating that there is a slight deviation between the predictions and the actual values (Fig 5). This can possibly be rectified by increasing the training sample size as generally, it is inferred that using sufficient data set for predictive model construction can lead to better accuracy [66].

### DNA structure prediction and docking

**Fig 6:**
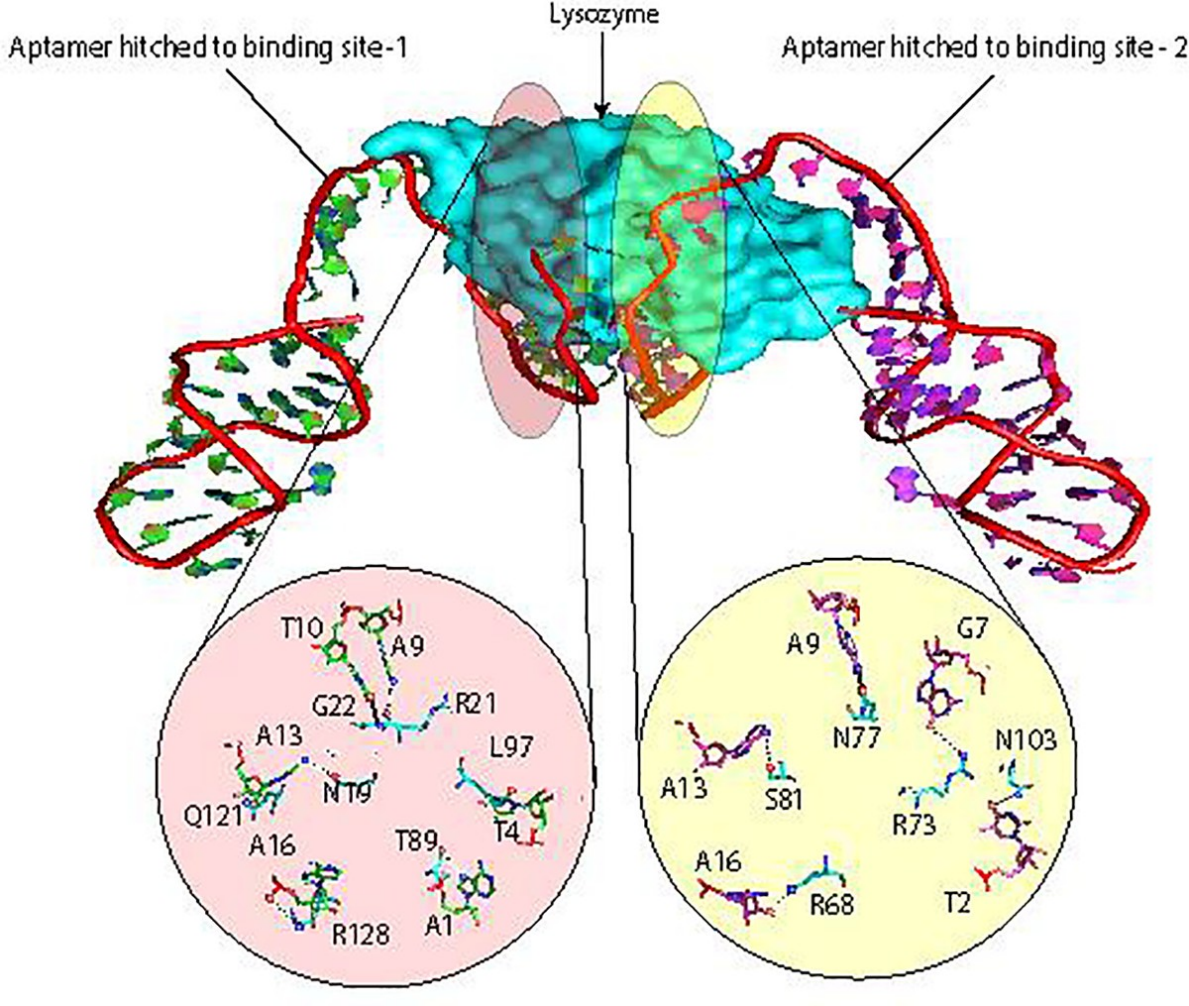
Lysozyme with two anti-Lys aptamers bound to its two binding sites.

The predicted 3-D structure of the aptamer obtained from RNA Composer (S1 Fig in S1 File) and minimized using NAMD (S2 Fig in S1 File) suggested that the 5’-ter of the aptamer wrapped the protein while the 3’-ter produced a hairpin loop that made no contact with Lys (Fig 6), indicating the absence of any role for 3’-ter in the Lys binding. In order to identify the appropriate mode among the two hypothesized for the aptamer, the aptamer was docked with lysozyme and the top 10 Lys-Apt models were re-docked with another aptamer molecule to get another set of top 10 model Apt-Lys-Apt models. Among the 100 resultant models, the one without congruent sites with the highest dock score was considered. The successful model possessed a dock score of -267.68 and an RMSD of 62.64 while the second binding site exhibited a dock score of -369.82 and an RMSD of 45.50. The bonds involved in both the binding sites were uncovered, as shown in Fig 6. The aptamer produced very few interactions with the protein side chain which is expected as the aptamer screening SELEX process is performed with the protein in its native structure and presents very few side-chains facing the exterior of the protein.

The aptamers bound to both the sites had free 3’-terminals which indicated the lack of any role for 3’-ter in the protein interaction. This suggested that mode 1 is not probable and mode 2 is more prospective. In order to probe the same (neighbour-aptamer interaction), the free 3’- terminal of the aptamer which formed a hairpin was severed. This severed terminal was then subjected to hairpin binding energy and heterodimer formation analysis as a higher binding energy for the heterodimer (neighbour aptamer interaction) would indicate its spontaneity over hairpin formation. Subsequently, the binding energy of the 3’-ter hairpin (-3.81 Kcal/mol) calculated using Vfold2D web server (S3 Fig in S1 File) was found to be lesser than the binding energy (-11.70 Kcal/mol) of the 3’-ter double strand (neighbour-aptamer interaction) calculated using VfoldCPX web server.

**Fig 7:**
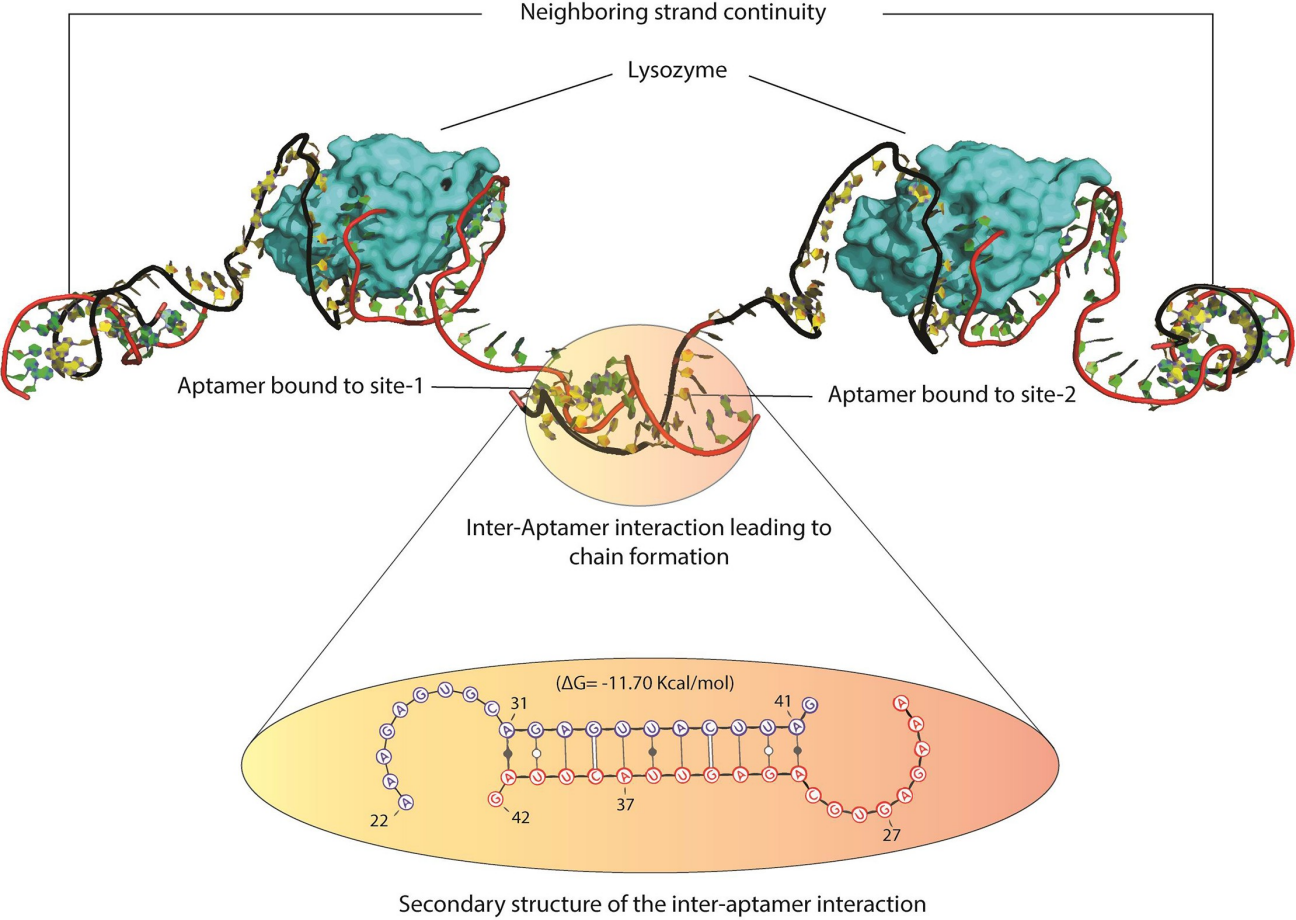
Representation of the polymeric chain highlighting the interacting base pairs in the inter-aptamer interaction.

This exercise exemplified Mode-2 as the probable cause for formation of aggregates with one Apta-Lys-Apta complex acting as a monomeric unit. The potential 3-D structure of this polymeric complex was then constructed by merging the 3-D structure output of 3’-ter interactions obtained from RNAcomposer with the previously obtained Apta-Lys-Apta complex as shown in Fig 7. The accuracy of the docked poses hold more room for improvement since the rigid docking strategy followed here is less precise due to the lack of conformational flexibility of the lysozyme and aptamer [67]. It further disregards the solvent and entropic contributions to the binding free energy and hence these calculations are also required for further accuracy [68].

This work focuses on the detection of Lys, a significant biomarker for many diseases however it is not specific to any particular ailment. Nevertheless, we believe that this research holds a lot of promise for custom designing future aptamers for physiologically more significant biomarkers. The significance of finding multiple binding sites on a protein for the same aptamer like the current study, is not just limited to aggregation assay development. It can also be extended to development of novel sandwich assays and innovative in-vivo aptamer-based treatment procedures. The LOD achieved by this method through normal photography prevails in the mM range because of the invisibility of micro-aggregates to the naked eye. A better LOD can be achieved through texture analysis of photographs of microscopic images or with sensitive turbidimetric instruments. In that perspective, point-of-care capabilities with low LODs can be realized in subsequent investigations through the development of compact optical setups for photomicrography or ultra-portable turbidimetric equipment. Additionally, the results obtained from the neural network exhibited a slight deviation from the expected values. This can be rectified with a higher sample size. However, the vagueness that surrounds the impact of the dataset size on prediction accuracy makes it difficult to say precisely when a dataset is big enough to provide accurate results, which makes this another future direction for study [44]. Finally, upcoming studies can be geared towards solidifying the underlying principle and augment the current bioinformatics studies with crystallographic data. We believe that these are the key areas that can steer future research in this highly promising direction.

## Conclusions

Herein, we have described a novel assay technique to quantify Lys via texture analysis and neural networking. Further, we probed into the molecular workings of the aptamer-analyte interaction responsible for the aggregation property, through bioinformatics tools. The significance of this study lies on two fronts- the bioinformatics analysis and the assay technique. The bioinformatics part of the article sheds light to the mode of aptamer-analyte interaction, a mechanism that can be useful for rational re-design/improvement of screened aptamers. This workflow presents many untapped aptamer-analyte properties that could be exploited for novel assay development. On that pretext, this article explores the aggregation property of the Ellington-group aptamer and exploits it for quantification using a beadless aptamer aggregation assay. On the assay front, the texture-based quantification reported here further expands the ever-growing repertoire of novel quantification techniques. Quantification of aggregation is generally facilitated by complex laboratory equipment thereby making them unfit for point-of-care and low-cost applications. Nonetheless, this article circumvents the problem by using a smartphone and a computer leading to low assay cost and higher point-of-care capability. The limit of detection of this method was experimentally found to be 1 mM and the volume of sample used for detection was 10 μl for 25–400 mM range and 100 μl for 1–20 mM range.

## Supporting information

S1 File (DOCX)
