# Supplementary material for: An apta-aggregation based machine learning assay for rapid quantification of lysozyme through texture parameters
Source: PLoS One. 2021 Mar 8;16(3):e0248159. doi: 10.1371/journal.pone.0248159 (PMC7939288; doi:10.1371/journal.pone.0248159)
Supplement: S1 File — (DOCX) [file pone.0248159.s001.docx]

Full title: An apta-aggregation based machine learning assay for rapid quantification of lysozyme through texture parameters

Short title: A new aptamer-based analytical method

Manoharan Sanjay^1^, Kumar Gaurav^2^, Maria Jesus Gonzalez-Pabon^1^, Julio Fuchs^1^, Susan Mikkelsen^3^, Eduardo Corton^1*^

^1^ Biosensors and Bioanalysis Laboratory (LABB). Department of Biological Chemistry and IQUIBICEN-CONICET. Exact and Natural Sciences Faculty (FCEN), University of Buenos Aires (UBA), Argentina

^2^ Department of Biosciences and Bioengineering. Indian Institute of Technology Guwahati. Guwahati, Assam, India

^3^ Department of Chemistry. University of Waterloo. Waterloo, Ontario, Canada

^*^ Corresponding author

E-mail: [eduardo@qb.fcen.uba.ar](mailto:eduardo@qb.fcen.uba.ar)

**
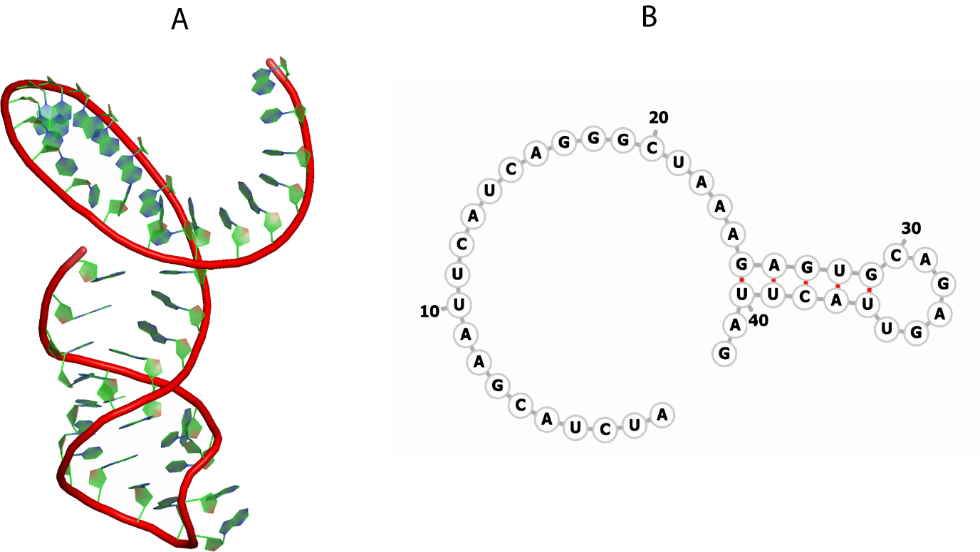
**

**S1 Fig. A) Tertiary structure of RNA aptamer B) Secondary structure of the RNA aptamer.**


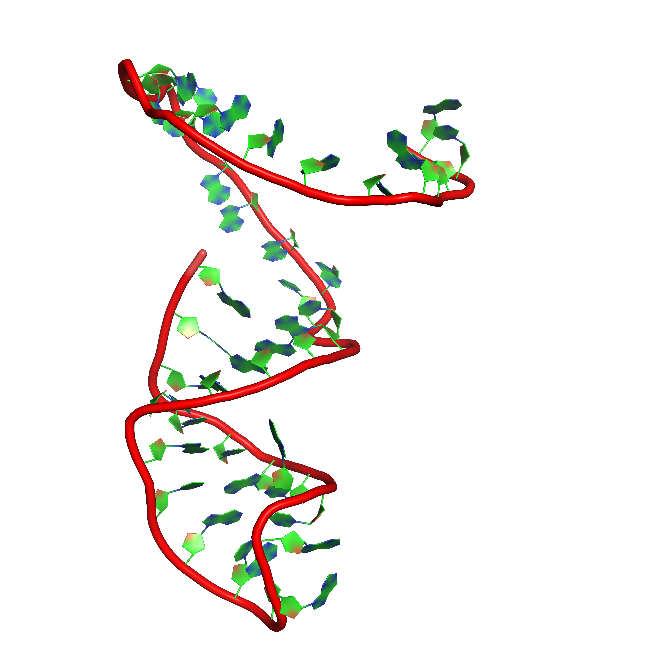


**S2 Fig. NAMD minimized tertiary structure of aptamer.**


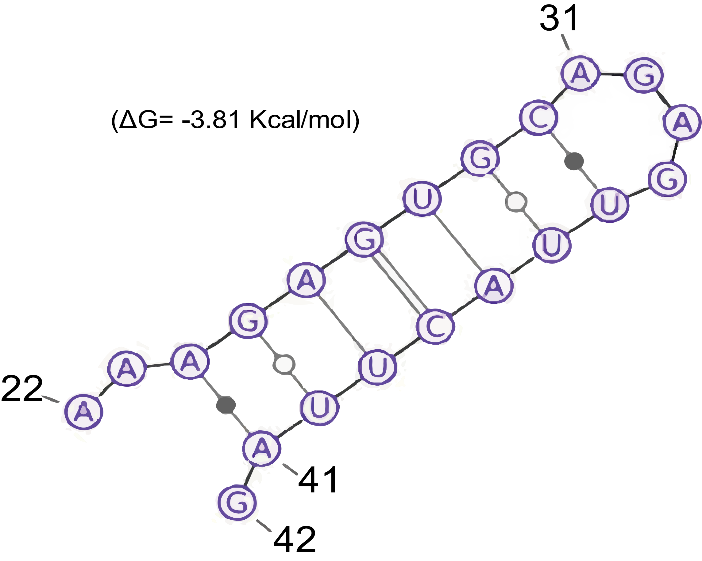


**S3 Fig. Hairpin structure of the RNA aptamer and its binding energy obtained from Vfold2D.**

**S4 Fig. Linear fit of aptamer volume versus aggregate area.** The minimal volume of aptamer to be used in the assay was calculated as 0.66 µL using the formula, Limit of Detection (LOD) = 3.3 (Sy/S)

Where, standard deviation of the response (Sy) of the curve and the slope of the calibration curve (S). 0.66 µL contains 0,0165 picomoles of aptamer, that will be the minimal amount of aptamer needed for one analysis in this condition (fixed lysozyme concentration).

Some analysis of the curves presented in the Fig. 4 are shown in tables S1 and S2. Extraction of calibration analytical data from these plots is not beneficial on most occasions and hence led to the implementation of supervised machine learning to obtain lysozyme concentration from the plot data. Nevertheless, some useful information concerning the sensitivity of the slopes of linear fitting at the lowest concentrations of each set have been included here for the extensive reference of the reader.

S1 Table. Sensitivities for the different parameters presented in Fig 4, Set-A. Linear range was found between 25-100 mM, and its corresponding sensitivity was expressed as response/[lysozyme]mM.

| Parameter | Area | ASM | Contrast | Correlation | IDM | Entropy |
| --- | --- | --- | --- | --- | --- | --- |
| R^2^ | 0.98 | 0.91 | 0.99 | 0.89 | 0.95 | 0.98 |
| Sensitivity | 65.65 | 16.06 | -0.456 | -3E-06 | 0.008 | 0.0024 |

S2 Table. Sensitivities for the different parameters presented in Fig 4, Set-B. Linear range was found between 1-4 mM.

| Parameter | Area | ASM | Contrast | Correlation | IDM | Entropy |
| --- | --- | --- | --- | --- | --- | --- |
| R^2^ | 0.93 | 0.91 | 0.94 | 0.74 | 0.91 | 0.98 |
| Sensitivity | -5440.3 | 0.0004 | -15.26 | -3E-05 | 0.021 | -0.123 |
